# Establishing an Anaesthesia and Intensive Care partnership and aiming for national impact in Tanzania

**DOI:** 10.1186/s12992-016-0144-1

**Published:** 2016-03-18

**Authors:** Mpoki Ulisubisya, Henrik Jörnvall, Lars Irestedt, Tim Baker

**Affiliations:** Society of Anaesthesiologists of Tanzania, PO Box 65588, Dar es Salaam, Tanzania; Mbeya Zonal Referral Hospital, Mbeya, Tanzania; Department of Anaesthesia, Intensive Care and Surgical Services, Karolinska University Hospital, 171 76 Stockholm, Sweden; Department of Physiology and Pharmacology, Section for Anaesthesiology and Intensive Care Medicine, Karolinska Institute, Stockholm, Sweden; Global Health – Health Systems and Policy, Department of Public Health Sciences, Karolinska Institute, Stockholm, Sweden

**Keywords:** Anaesthesia, Critical care, Health services, Quality of health care, Capacity building, Developing countries, Africa, Tanzania, Global health, Cooperative behaviour

## Abstract

Anaesthesia and Intensive Care is a neglected specialty in low-income countries. There is an acute shortage of health workers – several low-income countries have less than 1 anaesthesia provider per 100,000 population. Only 1.5 % of hospitals in Africa have the intensive care resources needed for managing patients with sepsis. Health partnerships between institutions in high and low-income countries have been proposed as an effective way to strengthen health systems. The aim of this article is to describe the origin and conduct of a health partnership in Anaesthesia and Intensive Care between institutions in Tanzania and Sweden and how the partnership has expanded to have an impact at regional and national levels.

The Muhimbili-Karolinska Anaesthesia and Intensive Care Collaboration was initiated in 2008 on the request of the Executive Director of Muhimbili National Hospital in Dar es Salaam. The partnership has conducted training courses, exchanges, research projects and introduced new equipment, routines and guidelines. The partnership has expanded to include all hospitals in Dar es Salaam. Through the newly formed Life Support Foundation, the partnership has had a national impact assisting the reanimation of the Society of Anaesthesiologists of Tanzania and has seen a marked increase of the number of young doctors choosing a residency in Anaesthesia and Intensive Care.

## Background

Anaesthesia and Intensive Care is a neglected specialty in low-income countries (LICs). There is an acute shortage of health workers – several low-income countries have less than 1 anaesthesia provider per 100,000 population [1]. In Malawi and Zambia 95 and 78 % of anaesthesia providers are non-physicians respectively [2, 3]. Less than one in fifteen hospitals in Uganda have the facilities to deliver safe anaesthesia for Caesarean section [4]. There is a profound lack of Intensive Care Unit (ICU) capacity in LICs: Uganda has 1.0 ICU bed per million population [5]. Of hospitals in Africa, only 1.5 % have the intensive care resources needed for managing patients with sepsis [6].

The state of Anaesthesia and Intensive Care in Tanzania, a low-income country in East Africa, is similar to other LICs. There are 22 anaesthesiologists for a population of 47 million (ratio 0.05: 100,000) [7]. Most anaesthesia is carried out by non-physician anaesthetists, many of whom lack formal training and qualifications [7]. The quality of anaesthesia is sub-optimal and varies greatly between providers [8]. Very few hospitals in Tanzania have intensive care units (ICUs), and there are structural barriers for care of the critically ill with deficiencies in infrastructure, human resources, training, and clinical routines [9].

To put these figures in perspective, in Sweden, the 2536 anaesthesiologists are more numerous than any other hospital specialty, and have a ratio of one to 3500 population [10]. A similar ratio is seen in the UK, which has one physician anaesthetist for every patient undergoing surgery, and in the USA with a workforce of 70,000 anaesthesia providers [1]. ICU beds are 9 % of total bed capacity in the USA and offer advanced organ support by specialist trained staff [11]. It is estimated that expenditure on Intensive Care is 13.3 % of total hospital expenditure in the USA [12].

In recent years it has been recognised that anaesthesia and intensive care have global public health significance [11, 13–15]. Over 230 million operations are performed globally every year, and 14 % of the overall global burden of disease is treatable by surgery [16, 17]. Most surgical illness is in poor countries and yet only 3.5 % of operations are conducted there [17]. Five billion people lack access to safe, affordable surgery and anaesthesia [18]. The burden of critical illness is highest in LICs with 90 % of trauma deaths, the majority of maternal deaths and 90 % of paediatric deaths in such settings [14, 19]. LIC have the greatest burden of sepsis [20]. Surgery, Anaesthesia and Intensive Care have previously been criticised as being too expensive for resource-limited settings, but recent studies have shown a favourable cost-effectiveness [18].

Health partnerships between institutions in High-income countries and LICs have been proposed as an effective way to strengthen health systems [21]. There is a scarcity of information about such partnerships within Anaesthesia and Intensive Care. One recent account of a partnership in Uganda describes an improvement in surgical and perioperative care capacity [22], and an education and mentorship project in Rwanda reports improvements in clinical practice [23]. Few partnerships describe an expansion beyond the level of local hospitals and training institutions. The aim of this article is to describe the origin and conduct of our health partnership in Anaesthesia and Intensive Care between institutions in Tanzania and Sweden and how the partnership has expanded to have effects at regional and national levels.

## Origin of the partnership

The partnership was initiated in 2008 by the then Executive Director of Muhimbili National Hospital (MNH) in Dar es Salaam, Prof Leonard Lema. He recognised that Anaesthesia and Intensive Care was the weakest specialty in the hospital and that a health partnership could lead to improvements. His vision was to use the partnership to encourage young doctors to choose Anaesthesia and Intensive Care as their specialty and to improve the quality of services at MNH. Through his contact at Karolinska University Hospital, Prof Jan Lindsten and subsequently Associate Prof Lars Irestedt, Prof Lema connected the departments of Anaesthesia and Intensive Care at the two hospitals. A needs assessment was conducted by the anaesthesiologists Dr Ulisubisya Mpoki at MNH and Dr Tim Baker at Karolinska which resulted in a formal link and the creation of the Muhimbili-Karolinska Anaesthesia and Intensive Care Collaboration (MKAIC).

From the outset, MKAIC aimed to be a strong, long-term, sustainable partnership. Guidelines on establishing health partnerships from the Tropical Health and Education Trust (THET) (www.thet.org) were followed, including setting up Link Committees in both hospitals, with Dr Mpoki and Dr Baker as the first two coordinators, establishing communication methods and agreeing joint goals. The goal of MKAIC is to “build cross-cultural understanding and partnership between Karolinska University Hospital in Sweden and Muhimbili National Hospital in Tanzania with the aim of improving knowledge and skills and strengthening healthcare services in Anaesthesia and Intensive Care” (www.mkaic.org).

## Activities

MKAIC’s activities since 2008 can be seen in Table 1.

**Table 1:** MKAIC’s activities and achievements 2008-2015

Activity	Details	Achievements
Training	Training courses in Dar es Salaam	9 training courses held
		350 doctors & nurses have been trained in Anaesthesia and Intensive Care
		Knowledge levels have increased by 30 % in pre-post course tests
Exchanges	Staff exchanges between hospitals in Dar es Salaam and Karolinska in Stockholm	17 doctors and nurses from Dar es Salaam have had placements at Karolinska
		29 doctors and nurses from Stockholm have been to hospitals in Dar es Salaam
Checklists	Designing and introducing clinical checklists for Obstetric Anaesthesia, Intensive Care and Post-operative Care introduced at MNH	Checklists for Obstetric Anaesthesia, Intensive Care and Post-operative Care introduced at MNH
Research	Investigate the optimal ways for running Anaesthesia and Intensive Care in MNH and in other low-resource settings	4 published articles in peer-reviewed journals
		2 manuscripts undergoing review
		2 manuscripts ongoing
Equipment	Introduce equipment that is lacking for Anaesthesia and Intensive Care	125 pulse oximeters, other equipment and disposable items introduced to hospitals in Tanzania
Regional activities	Advocacy for Anaesthesia and Intensive Care	Secured the support of the Regional Medical Officer in Dar es Salaam
	Regional support	Established contact with all regional and district hospitals in Dar es Salaam
	Involvement of other hospitals	Staff from other hospitals involved in training and exchanges
National activities in Tanzania & Sweden	Advocacy for Anaesthesia and Intensive Care	Anaesthesia and Intensive Care advocacy at the Ministry of Health and Social Welfare.
	National support	Secured written support from the Permanent Secretary
	Support for SATA	Assisted the reanimation of SATA and the first two National Scientific Conferences in Tanzania
	Encourage more young doctors to choose Anaesthesia and Intensive Care	Collaboration with MUHAS
		Seen an increased number of residents in Anaesthesia and Intensive Care from one to nine in 2014, including one sponsored by Life Support Foundation

*MNH* Muhimbili National Hospital, *MUHAS* Muhimbili University of Health and Allied Sciences, *SATA* Society of Anaesthesiologists in Tanzania

### Training

Training courses have been held annually at Muhimbili in Obstetric Anaesthesia & Care for the Critically Ill Mother, Paediatric Anaesthesia & Care for the Critically Ill Child and Intensive Care. The interactive courses are for between 30-50 participants and are taught by a mixed faculty from MNH and Karolinska. The participants are nurses, non-physician clinicians and physicians from various departments. Training focuses on the management of critically ill patients and patients undergoing surgery in resource limited environments. The principles of teamwork, communication, systematic preparation and respect for the patients and other health-workers are emphasised throughout the course. The training is based on the ABC (Airway, Breathing, and Circulation) model of acute healthcare, and aims to standardise ABC care so that all health-worker cadres are trained in the same principles.

### Exchanges

Staff from MNH spend between 2 weeks and 3 months at Karolinska, sharing ideas with the local staff and experiencing Anaesthesia and Intensive Care in a high-resource milieu. The aim is exposure to the same guiding principles as in the training courses, rather than the advanced treatments sometimes offered at Karolinska. MKAIC is a partnership that acts in both directions. With our reciprocal model, staff from Karolinska have also spent time at MNH, bedside teaching and sharing ideas. Swedish staff gain experience of Anaesthesia and Intensive Care in a low-resource milieu, learning about global health, contrasting disease panoramas and cultures and how to conduct healthcare with fewer human resources and support structures.

### Routines and checklists

We have identified standardised preparation and routines for Anaesthesia and Intensive Care as a weak point in patient care at MNH. To alleviate this, teams at MNH and Karolinska have designed checklists for obstetric anaesthesia, intensive care and for post-operative care at MNH. Large posters have been set up on the walls of clinical areas and paper versions introduced for use for individual patients.

### Research

We have conducted several collaborative audit and research projects to systematically investigate the optimal ways for running Anaesthesia and Intensive Care in MNH and in other low-resource settings. As of April 2015, four papers have been published [8, 9, 24, 25], two are under review and a further two are in the manuscript phase.

### Equipment

Pulse oximetry was identified early on in the collaboration as the equipment that had the greatest potential to improve care. Through a collaboration with Lifebox Foundation (www.lifebox.org) we have introduced 125 pulse oximeters to hospitals in Dar es Salaam and throughout Tanzania. Course literature has been provided by a donation from the Association of Anaesthetists of Great Britain and Ireland. Other equipment and disposable items have also been supplied from Sweden, as requested by the MKAIC MNH Committee.

## Expanding to a regional level

Patients are referred to MNH from the district and regional hospitals in Dar es Salaam. To improve the quality of referrals, and reduce the numbers that need referral, the regional health authorities have worked to improve the quality of care at all hospitals in the city. As part of this drive, MKAIC expanded in 2012 to involve staff from all hospitals in Dar es Salaam. This was approved by the Regional Medical Officer and contact was made with the departments of Anaesthesia and Intensive Care at Amana, Temeke and Mwanyanamala hospitals, as well as the smaller district hospitals and Muhimbili Orthopaedic Institute. The hospitals could send staff to all MKAIC courses and on MKAIC exchanges and equipment was delivered. Activities were coordinated with regional efforts to improve Anaesthesia and Intensive Care, under the guidance of the Comprehensive Community Based Rehabilitation Hospital (CCBRT) in Dar es Salaam.

## Expanding to a national level

To achieve our aim of improving Anaesthesia and Intensive Care in Tanzania, we have needed to act nationally. To facilitate this, we have established the Life Support Foundation (www.lifesupportfoundation.org) in Sweden. We have held the first Swedish workshop on Global Anaesthesia and Intensive Care at the Swedish Society of Anaesthesia & Intensive Care (SFAI) national conference in September 2015.

We have worked with the national training institution in Tanzania, Muhimbili University of Health and Allied Sciences (MUHAS), to promote specialist training in Anaesthesia and Intensive Care. One resident doctor at MUHAS has been sponsored by Life Support Foundation. The research projects have been carried out in collaboration with MUHAS. The dormant Society for Anaesthesiologists in Tanzania (SATA) was reanimated by Dr Mpoki with the assistance of Life Support Foundation, with a newly formed committee and their first National Scientific Conferences in 2014 and 2015. We have initiated a database of international efforts to improve Anaesthesia and Intensive Care in Tanzania, as a first step to coordinating and developing synergy between the projects. Pulse oximetry training in several regions of the country is planned for 2016.

We have had several meetings with senior officials in the Ministry of Health and Social Welfare, to advocate the vital importance of Anaesthesia and Intensive Care and to secure support for our collaboration. The Permanent Secretary has written to us to give his support. At the start of the 2014-2015 academic year, thirteen residents registered to the specialist training programme in Anaesthesia and Intensive Care at MUHAS, a marked increase from previous years (one in 2013, none in 2012, two in 2011).

## Strengths of our partnership

A great strength of MKAIC is that it was initiated by the partner in the LIC. Doctors at MNH saw the need for improving Anaesthesia and Intensive Care and approached Karolinska. MKAIC is embedded in the Anaesthesia and Intensive Care departments at both hospitals, and has been enthusiastically supported by departmental heads. There is a clear and pressing need for improvements in Anaesthesia and Intensive Care at MNH, and a lack of other initiatives working in this field.

MKAIC has always had a long-term, sustainable focus, which is essential when working with system-wide capacity building. There are no quick fixes or silver bullets – improving services inevitably takes time. Our expansion from a single hospital focus to regional and national involvement, and securing support from hospital leaders and policy makers has made MKAIC robust and embedded within Tanzania’s health system.

## Threats to our partnership

There have been several challenges to MKAIC, and threats to the partnership’s success. It has not yet been possible to secure long-term funding, either from within Tanzania or from large international grants. Funding has been on an ad hoc basis; each project and activity has raised funds needed for running costs. All staff involvement in MKAIC has been on a pro bono basis, and MKAIC has had a “no per-diem” policy for the training courses and exchanges. There is a hope that sustainable funding will be secured in 2016.

Communication between the committees has not always worked optimally. We have not managed to set up regular video conferencing and email contact has been sporadic. Telephone calls have been the best communication method, but are costly. We have wanted to have long-term exchanges of staff between MNH and Karolinska, but have not been able to organise this logistically or financially. There have been several personnel changes in the department of Anaesthesia and Intensive Care during the 7 years of our partnership, which has affected continuity and communication.

## Effects of our partnership

Table 1 shows what we have achieved. There are many anecdotal accounts of improved Anaesthesia and Intensive Care services at MNH and at the other hospitals in Dar es Salaam (see case and feedback boxes). We have improved acute treatments of deranged physiological parameters on the ICU at MNH [26]. Knowledge levels have been raised by 30 %, as measured by pre/post training course tests (unpublished results). We have raised the profile of Anaesthesia and Intensive Care in Tanzania, assisted a reanimation of the Society of Anaesthesiologists in Tanzania and seen a marked increase in the number of resident doctors choosing Anaesthesia and Intensive Care.

**Table:** 

**Case** A baby boy was delivered by Caesarean Section at Muhimbili. He was carried to the resuscitation table by the anaesthetic nurse. The baby was blue and wasn’t breathing. The nurse had been on an MKAIC training course the week before and put into practice the simple ABC rules that she had been taught. Using a bag-and-mask that had been brought from Karolinska, she was able to inflate the baby’s lungs and re-oxygenate the blood. After 10 minutes the baby started breathing by himself and began crying soon after.

**Table:** 

**Feedback from a Nurse Anaesthetist 6 months after an MKAIC course** “Actually, on my side I’m doing quite a lot and I’m very proud of your teaching which you gave us, especially anaesthesia for caesarean section by using spinal. Since I started to practice this there is no complication. I’m very very very happy!”	

## Future plans

We plan to continue this long-term health partnership in Anaesthesia and Intensive Care. We want the improvements to be sustainable and embedded in the health system. The partnership will continue its expansion nationally, to include more hospitals in Tanzania. We will encourage and assist SATA in playing a vital role in Anaesthesia and Intensive Care in Tanzania, increasing membership of the association and its activities throughout the country. We would like the Ministry of Health and Social Welfare to make Anaesthesia and Intensive Care a priority area for improvement, leading to national guidelines and standards for safe anaesthesia, the recognition of anaesthesia staff and increased human resources. We aim to expand our research activities and conduct pragmatic implementation research about the optimal ways of organising and managing Anaesthesia and Intensive Care when resources are limited. We would also like to expand the partnership between Muhimbili and Karolinska to include other specialties, and have already begun this process with Obstetric and Paediatric projects.

## Conclusion

We have described the process for initiation and conduct of a health partnership in Anaesthesia and Intensive Care between institutions in Tanzania and Sweden. A partnership that has expanded from its initial localised focus, to aim for regional and national impact.

## Abbreviations

ABC: Airway, Breathing, Circulation
CCBRT: Comprehensive Community Based Rehabilitation in Tanzania
ICU: intensive care unit
LIC: low-income country
MKAIC: Muhimbili-Karolinska Anaesthesia and Intensive Care Collaboration
MNH: Muhimbili National Hospital
MUHAS: Muhimbili University of Health and Allied Sciences
SATA: Society of Anaesthesiologists of Tanzania
SFAI: Swedish Society of Anaesthesia & Intensive Care
THET: Tropical Health and Education Trust
